# Exposure to second-hand smoke and risk of lung cancer among Iranian population: A multicenter case-control study

**DOI:** 10.1371/journal.pone.0306517

**Published:** 2024-07-10

**Authors:** Fereshte Lotfi, Hamideh Rashidian, Maryam Hadji, Elham Mohebbi, Maryam Marzban, Ahmad Naghibzadeh-Tahami, Eero Pukkala, Paolo Boffetta, Arash Etemadi, Kazem Zendehdel

**Affiliations:** 1 Cancer Research Center, Cancer Institute of Iran, Tehran University of Medical Sciences, Tehran, Iran; 2 Department of Medical and Surgical Sciences, University of Bologna, Bologna, Italy; 3 Health Sciences Unit, Faculty of Social Sciences, Tampere University, Tampere, Finland; 4 Lombardi Comprehensive Cancer Center, Georgetown University, Washington, DC, United States of America; 5 Department of Public Health, School of Public Health, Bushehr University of Medical Science, Bushehr, Iran; 6 Statistical Genetics Lab, QIMR Berghofer Medical Research Institute, Brisbane, QLD, Australia; 7 Health Services Management Research Center, Institute for Futures Studies in Health, Kerman University of Medical Sciences, Kerman, Iran; 8 Department of Biostatistics and Epidemiology, Kerman University of Medical Sciences, Kerman, Iran; 9 Finnish Cancer Registry—Institute for Statistical and Epidemiological Cancer Research, Helsinki, Finland; 10 Stony Brook Cancer Center, Stony Brook University, Stony Brook, NY, United States of America; 11 Metabolic Epidemiology Branch, Division of Cancer Epidemiology and Genetics, National Cancer Institute, Rockville, Maryland, United States of America; Shahid Beheshti University of Medical Sciences School of Dentistry, ISLAMIC REPUBLIC OF IRAN

## Abstract

**Objective:**

Despite the implementation of the WHO Framework Convention on Tobacco Control (FCTC) program in Iran, the regulation of second-hand smoke (SHS) exposure—an often-overlooked hazard—, still requires improvement. We employed a multi-center case-control study to investigate the association between exposure to secondhand smoke (SHS) from various tobacco products (cigarettes, water-pipes, pipes, and chopogh), opium use, and the risk of lung cancer.

**Method:**

We included 627 lung cancer cases and 3477 controls. Exposure to SHS tobacco and SHS opium was collected through a questionnaire. We used mixed-model logistic regressions to estimate odds ratios (ORs) and 95% confidence intervals (CI).

**Result:**

Among the overall population exposed to second-hand tobacco smoke (SHTS), the odds ratio (OR) compared to those never exposed was 1.35 (95% CI: 1.08–1.71). Never smokers who were ever exposed to second-hand tobacco smoke (SHTS) had 1.69-fold risk of lung cancer compared to those who were never exposed (95% CI: 1.13–2.52). Exposure to SHTS between 2–3 per day (OR = 2.27, 95% CI: 1.13–4.53) and more than three hours per day (OR = 2.29, 95% CI: 1.20–4.37) can increase the risk of lung cancer compared with the no exposure group (P-trend <0.01). We did not observe any association between exposure to second-hand opium smoke (SHOS) and the risk of lung cancer, either in the overall population or among never-smokers.

**Conclusion:**

Our study estimates the impact of second-hand tobacco smoke (SHTS) on lung cancer risk in both the overall population and never-smokers. Additional studies are required to evaluate the association between exposure to second-hand smoke from opium and other type of tobacco, including water-pipe and the risk of lung cancer.

## Introduction

Tobacco smoking is indisputably known as a risk factor for lung cancer. Studies have demonstrated lung cancer has the highest proportion of smoking-attributable cases (about 80%) [1–3]. While tobacco smoking is considered a leading cause of lung cancer, 10–15% of lung cancer cases occurred among people who never smoked [4]. Initially, there was a lack of available information regarding the descriptive epidemiology of lung cancer among never smokers. Evidence about this gap beyond the air pollution and occupational risk factors such as radon and asbestos remain unrecognized until the 1980s [5]. Thereafter several epidemiological studies have demonstrated the role of passive smoking or second-hand smoke (SHS) as a risk factor for adverse health outcomes, including respiratory effects in both children and adults, coronary heart disease and, cancer [6–10].

Second-hand tobacco smoke (SHTS) encompasses “mainstream smoke” exhaled by a smoker after inhaling cigarette smoke, and “sidestream smoke” derived from the tip of a cigarette, cigar, or smoking pipe [11]. Given that sidestream smoke is generated at a lower temperature from incomplete combustion, it comprises carcinogens with thicker density compared to mainstream smoke [12]. In general, it is estimated that there are more than 60 carcinogens or compounds with strong evidence of carcinogenicity in cigarette smoke and most of them are present in sidestream too. Many of these compounds are classified in groups 1 and 2A (carcinogenic and probably carcinogenic to humans) by the International Agency for Research on Cancer (IARC) [13].

Epidemiological findings on passive smoking indicated an association between SHTS and the risk of developing lung cancer [14–17]. Although, this association does not appear to receive much attention in the low- and middle-income countries [18]. In Eastern Mediterranean Region (EMRO) countries—where tobacco smoking is less common among women due to stigma and traditional cultural constraints—lung cancer is among the top 10 common cancer types in women [19]. In this region, 32% of women, a considerably higher percentage compared to the 22% of men, are exposed to second-hand smoke. Given that these women represent only 25% of the labor force, it is likely that a substantial amount of exposure occurs within their homes [20]. Additionally, it appears that pro-health policies related to waterpipe smoking in this region have lagged behind those for cigarette. The widespread misconception that water pipes are less harmful compared to other types of tobacco has led to water pipe smoking becoming the preferred choice of tobacco use in some of our provinces. The available information on waterpipe smoking in these regions raises concerns, particularly among young people. Consequently, measuring secondhand smoke exposure for this specific type of tobacco is essential [21–25].

In Southeast and East Asia, the proportion of lung cancer cases among never-smokers is notably higher than in other regions, ranging from 30% to 50% [26]. Given the significant prevalence of opium use in this region [27], it is plausible that exposure to second-hand opium smoke (SHOS) may contribute to this variation. Despite the widespread use of opium in countries such as Afghanistan and Iran, the impact of second-hand opium smoke (SHOS) on the risk of lung cancer has not been considered [28–30].

We aimed to use the data from a large multicenter case-control study in Iran and assess the association between exposure to second-hand opium smoke (Tariak) and second-hand tobacco, including cigarettes and waterpipes, pipe and chopogh, and the risk of lung cancer.

## Method

### Study population

We used the Iranian Study of Opium and Cancer (IROPICAN) data, a large multicenter case-control study conducted in 10 provinces of Iran from 2016 to 2020, that investigates the association between opium use and the risk of lung, colorectal, bladder, and head and neck cancers. We recruited 627 pathologically confirmed cases of the trachea (International Classification of Diseases for oncology third edition (ICD-O-3): C33) and bronchus and lung (C34) and 3477 healthy hospital visitor controls. Controls were frequently matched with the cases of several studied cancer types combined by sex, age (10-year intervals) and place of residence (capital city of the province/other) [31].

### Exposure measurement

Details about questionnaire content are provided elsewhere [32]. In brief, we collected data on opium, cigarette and water pipe use, status including second-hand smoking and several confounding factors. We collected data about the ever exposure to second-hand tobacco including cigarettes, water-pipe, pipe and chopogh (a special pipe) and second-hand opium separately. Those who had daily exposure to SHTS during their lifetime were considered as ever exposed persons. A regular user was defined as a person who used the substances including cigarettes, waterpipe or opium at least once a week for six months. Those smokers not meeting the criteria for regular classification were designated as irregular smokers. The cumulative count of opium use was defined as the frequency of opium use multiplied by the duration of use. Cumulative cigarette smoking (pack-year) was calculated by multiplying the number of packs smoked in a day by the smoking duration (year). Cumulative water pipe smoking (head-year) was assessed by multiplying the number of heads used a day by smoking duration (year).

Additionally, detailed information about the number of daily hours and duration of exposure (month) to SHTS and SHOS was collected for ever exposed participants.

We used principal component analysis to determine the socioeconomic status of the participants [33]. Several components were used to evaluation of socioeconomic status which was mentioned elsewhere [32]. The response rates for cases and controls were 97% and 89%, respectively.

### Statistical analysis

We first performed the analyses on the entire case-control data set. In addition, to eliminate the confounding effect of active smoking we repeated the analyses among never smokers of any product (i.e., cigarette, water pipe and opium). Participants who were exposed to SHTS daily were considered as ever exposed. In order to adjust for the clustering effect of study centers, we used mixed model regression analyses to obtain odds ratios (ORs) with 95% confidence interval (CI) for the association between exposure to second-hand smoke and the risk of lung cancer. We adjusted for age (5 categories), gender, place of residence (capital city of the province /others) socioeconomic status (low/high), cigarette (pack-year), opium (frequency-year) and water pipe (head- year) use as potential confounders in all models. In order to investigate dose-response relationship between exposure to SHTS and lung cancer risk, we used both daily hours and duration of exposure (month) variables. We categorized users into three categories according to the 50th and 75th centile in the controls for hours and duration of exposure variable. We used STATA software for statistical analyses (Ver. 14.0, Stata Corp, College Station, Texas). The study was approved by the Ethics Committee of the National Institute of Medical Research Development (NIMAD) (Code: IR.NIMAD.REC.1394.027).

## Result

A total of 627 lung cancer cases with a mean age of 55.5 years and 3477 controls with a mean age of about 53 years were included in the study. The majority of cases and controls was men and older than 50 years old (Table 1). Although the overall proportion of women in the never-smokers group was higher compared to the total population. There was a significant association between exposure to SHTS and the risk of lung cancer in the overall population (OR = 1.35, 95% CI: 1.08–1.71) (Table 2). Also, among individuals who never smoked any product (i.e., cigarette, waterpipe, pipe, chopogh and opium), those who were ever exposed to second-hand tobacco smoke had a higher risk of lung cancer compared to the group that was never exposed (OR = 1.69, 95% CI: 1.13–2.52).

**Table 1:** Distribution of characteristics of the overall and never smokers among IROPICAN study participant.

	All		Never smokers^1^	
	Cases, n (%)	Controls, n (%)	Cases, n (%)	Controls, n (%)
	627(100)	3477 (100)	134 (100)	1943(100)
Age (years)				
30–39	14 (2.2)	257 (7.3)	7 (5.2)	164 (8.4)
40–49	74 (11.8)	559 (16.0)	17 (12.6)	323 (16.6)
50–59	204 (32.5)	1070 (30.7)	45 (33.5)	600 (30.8)
60–69	218 (34.7)	1092 (31.4)	40 (29.8)	591 (30.4)
≥ 70	117 (18.6)	499 (14.3)	25 (18.6)	265 (13.6)
Mean (SD)	55.58 (9.95)	52.92 (11.22)	54.40 (10.93)	52.41 (11.38)
Gender				
Men	482 (76.8)	2400 (69.0)	48 (35.8)	1069 (55.0)
Women	145 (23.1)	1077 (30.9)	86 (64.1)	874 (44.9)
Socioeconomic status^2^				
Low	271 (43.2)	974 (28.0)	58 (43.2)	559 (28.7)
moderate	225 (35.8)	1174 (33.7)	44 (32.8)	602 (30.9)
High	131 (20.8)	1329 (38.2)	32 (23.8)	782 (40.2)
Water-pipe smoking				
Non-user	553(88.5)	3221 (92.63)		
Irregular user	2 (0.31)	17 (0.48)		-
Regular user ^3^	72 (11.48)	239 (6.87)	-	-
Opium smoking				
Non-user	299 (47.6)	2870 (82.5)	-	-
Irregular user	19 (3.0)	138 (3.9)	-	-
Regular user ^4^	309 (49.2)	469 (13.4)	-	-
Cigarette smoking				
Non-user	222 (35.4)	2316 (66.6)	-	-
Irregular user	13 (2.0)	184 (5.2)	-	-
Regular user ^5^	392 (62.5)	977 (28.1)	-	-

Never smokers defined as people who had never smoked waterpipes, cigarettes, nor opium.

^2^ Tertile in control subjects was used as the dividing cut point.

^3^ Regular user: Smoking a head of water pipe per week for at least a 6-month consecutive period during the lifetime

^4^ Regular user: Using opium at least once a week for at least a 6-month consecutive period during the lifetime

^5^ Regular user: Smoking a cigarette per week for at least a 6-month consecutive period during the lifetime

**Table 2:** Adjusted odds ratios (ORs) with 95% confidence intervals (95% CI) of exposure to secondhand smoke and lung cancer in overall and among never smokers in Iran.

	All participants			Never smokers^1^		
	Cases	Controls	OR ^2^(95% CI)	Cases	Controls	OR ^3^(95% CI)
**Exposed to secondhand tobacco smoke** ^4^						
Never	365	2367	1.00	81	1392	1.00
Ever	166	751	1.35 (1.08–1.71)	42	401	1.69 (1.13–2.52)
**Exposure to secondhand tobacco smoke (Daily hours)**						
Never	365	2367	1.00	81	1392	1.00
<2	101	556	1.09 (0.86–1.39)	23	301	1.16 (0.73–1.83)
2–3	28	99	1.30 (0.86–1.96)	9	48	2.27 (1.13–4.53)
3>	37	96	1.30 (0.87–1.94)	10	52	2.29 (1.20–4.37)
P trend			0.09			0.01
**Exposure to secondhand tobacco smoke (year)**						
Never	365	2367	1.00	81	1392	1.00
= <20	105	464	1.10 (0.86–1.42)	21	237	1.39 (0.87–2.22)
21–27	22	114	1.37 (0.88–2.13)	6	64	1.66 (0.76–3.62)
> = 28	39	173	1.19 (0.67–2.11)	15	100	1.01 (0.23–4.39)
P trend			0.13			0.07
**Exposed to secondhand opium smoke** ^5^						
Never	365	2367	1.00	81	1392	1.00
Ever	21	76	1.17 (0.66–2.07)	2	29	1.00 (0.23–4.33)

Never smokers defined as people who had never smoked waterpipes, cigarettes, nor opium

^2^ Adjusted for age, gender, province of residence, pack-years of cigarette smoking, head-years of water-pipe smoking, cumulative count of opium and socioeconomic status

^3^ Adjusted for age, gender, province of residence, and socioeconomic status

^4^All participants, except for those exposed to second-hand opium and those exposed to both second-hand tobacco and opium

^5^ All participants, except for those exposed to second-hand tobacco and those exposed to both second-hand tobacco and opium

We found a positive association between daily hours of Second-hand tobacco smoke (SHTS) and risk of lung cancer among never smokers (P values for trend <0.01). Exposure to SHTS between two and three hours per day increased the risk of lung cancer compared to never exposed group (OR = 2.27, 95%CI: 1.13–4.53). The result was similar for the category of more than three hours of per day exposure to SHTS (OR = 2.29, %CI: 1.20–4.37). No association was found between the duration of exposure to SHTS and the risk of lung cancer. Also, we did not observe any association between lung cancer risk and exposure to second-hand opium smoke (SHOS) neither in the overall population (OR = 1.17, 95%CI: 0.66–2.07) nor among never smokers (OR = 1.00,95% CI:0.23–4.33). Likewise, examination of the joint effect didn’t show the interaction between regular smoking and exposure to SHTS (Result not shown in the Tables). Additionally, it was identified exposure to SHTS increased the risk of lung cancer among men (OR = 2.03, %CI: 1.04–3.97) and among individuals aged 60 or above (OR = 2.20, %CI: 1.25–3.87). No association was found between SHOS in term of age and gender distribution (Table 3).

**Table 3:** Adjusted odds ratios (ORs) with 95% confidence intervals (95% CI) of exposure to secondhand smoke and lung cancer in never smoker group by age and sex.

Never smokers^1^												
	Age						Gender					
	30–59			+60			Male			Female		
	Cases	Controls	OR^2^ (95% CI)	Cases	Controls	OR^2^ (95% CI)	Cases	Controls	OR^3^ (95% CI)	Cases	Controls	OR^3^ (95% CI)
**Exposed to secondhand tobacco smoke**												
Never	43	753	1.00	38	639	1.00	31	827	1.00	50	565	1.00
Ever	19	238	1.34(0.76–2.37)	23	163	2.20(1.25–3.87)	14	205	2.03(1.04–3.97)	28	196	1.57 (0.95–2.56)
**Exposed to secondhand opium smoke**												
Never	43	753	1.00	38	639	-	31	827	-	50	565	1.00
Ever	2	19	1.31(0.29–5.95)	0	10	-	0	9	-	2	20	1.22 (0.25–5.47)

## Discussion

We studied exposure to second-hand tobacco (cigarettes, water-pipe, pipe and chopogh) and second-hand opium smoke in a multi-center case control study on lung cancer in Iran. We observed increased risk of lung cancer in relation to SHTS overall and among never smoker group. Additionally, we found exposure-response trend for the number of hours per day of SHTS exposure and the risk of lung cancer in the never-smoker group. Our study revealed an increasing risk of lung cancer among men and those of advanced age who have been exposed to secondhand tobacco smoke (SHTS). However, there was no association between exposed to second hand opium and risk of lung cancer.

Our observations are in accordance with several lines of evidence that confirmed that exposure to SHTS increases the risk of lung cancer. According to studies, the risk of lung cancer can increase by around 25% in people exposed to second-hand smoke [19]. Results from the international lung cancer consortium (ILCCO) in 2014 one of the largest second-hand smoke studies, the meta OR from eighteen case-control studies comparing ever vs. never exposed to SHS was significantly elevated (OR = 1.34, 95% CI: 1.24–1.45). The risk of small cell incidence was more than 3-fold [14]. Brennan examined SHS exposure in adulthood and the risk of lung cancer among never smokers in 2004. The result of this study showed a positive association between long-term exposure to SHTS and the risk of lung cancer among never smokers (OR = 1.32, 95% CI: 1.04–1.66) [15].

In recent years, anti-smoking programs have been developed in high Human Development Index (HDI) countries and the tobacco industry targeted low and middle-income countries to sell their products. In addition, the popularity of some types of tobacco like waterpipe in the certain region like the Eastern Mediterranean Region (EMRO) has raised concern about those who don’t smoke but are passively exposed to smoking in these areas. In some Middle East countries, women do not smoke tobacco due to social and cultural norms. However, women and children are affected groups by second-hand tobacco smoke at home and workplaces [34, 35].

The dose-response relationship of SHTS and risk of lung cancer are compatible with other studies [14, 15, 36]. We also did not observe associations between the duration of secondhand smoke exposure and the risk of lung cancer. This issue possibly stems from the focus on the duration of consumption of tobacco and opium compared to the duration of exposure to second-hand smoke of these products in this study.

Exposure to second-hand opium smoke has greatly been ignored by countries with traditionally opium use like Iran, Afghanistan, and Pakistan. Test of air, surface and hair samples taken from the family of opium users by International Narcotics and Law Enforcement Affairs (INL) in Afghanistan showed that second-hand smoke of opium and even third-hand smoke could pose a major concern for children, particularly regarding their brain development but unfortunately, they did not evaluate the carcinogenicity of SHOS on Afghan population [30]. In our study, the sensitivity surrounding opium consumption, due to its illegality and people’s unwillingness to disclose information about their drug use, may explain why we didn’t observe any effects of SHOS.

We tested the interaction of SHTS exposure with active smoking and it was not significant However, the results from some studies on second-hand smoking suggest that the joint effects of active and passive smoking on lung cancer are consistent with a multiplicative model [14].

The strengths of this study include recruitment of a large sample with high response rate and recruitment of cases and controls from different provinces of Iran. Moreover, using hospital visitor controls which were shown more suitable option for this study as well as well-trained interviewers are another advantage of IROPICAN study [37]. However, this study had some limitations. Given that the primary focus of the IROPICAN study was on opium use, the assessment of exposure to second-hand tobacco smoke (SHTS) and second-hand opium smoke (SHOS) was considered of lower priority. As a result, the measurement of second-hand smoke exposure was not conducted in the most precise manner due to the inadequacy of the questions. Also, studies with a concentration on the assessment of various SHS aspects including duration of exposure at home, work, or in public places can measure exposure rate more precisely. Given the nature of our data collection procedure, recall bias cannot be ruled out. It is expected the cases recall their exposure more than control group. Given these assumptions, our result would probably overestimate the association. Another limitation in the present study was the low sample size of never smoker lung cancer cases, therefore we could not assess the association by lung cancer histology. It should be acknowledged that some individuals categorized as “never exposed” in the model may have experienced occasional exposure, which has the potential to result in an underestimation of the impact of secondhand smoke (SHS) exposure on lung cancer. Additionally, the limited sample size in this subgroup, may have posed a challenge in identifying any association between second-hand opium exposure and age or gender distribution.

Although consumption of opium is considerably high in Iran, we could not find any association between lung cancer and second-hand opium use. First, opium users also usually smoke tobacco and evaluation of the pure effect of opium smoke is complicated. On the other hand, Illegalizing and stigmatizing opium use are important obstacles to collecting data from users or passively exposed groups. Though, designing a larger study and using a comprehensive questionnaire along with biomarker validation is required for a full assessment of this association.

In conclusion, the objective of this investigation was to examine the association of secondhand smoke (SHS) of tobacco (cigarettes, water-pipe, pipe and chopogh) as well as second hand opium on individuals who have never smoked. Our findings indicate that SHTS increases the risk of lung cancer among the Iranian population. However, it is important to note that our results might underestimate the true risk of second-hand smoke on lung cancer. Further studies are required to verify the association between second-hand smoking and the risk of lung and other tobacco-related cancers in the Iranian population, particularly among women. Additionally, it’s essential to design a questionnaire to measure second-hand opium smoking in this hotspot region. It is also important to assess exposure to waterpipe smoking, a re-emerging virulent strain in the tobacco epidemic.
